# Methylation of CHFR sensitizes esophageal squamous cell cancer to docetaxel and paclitaxel

**DOI:** 10.18632/genesandcancer.46

**Published:** 2015-01

**Authors:** Tianyang Yun, Yang Liu, Dan Gao, Enqiang Linghu, Malcolm V. Brock, Dongtao Yin, Qimin Zhan, James G. Herman, Mingzhou Guo

**Affiliations:** ^1^ Department of Thoracic Surgery, Chinese PLA General Hospital, Beijing, China; ^2^ Department of Gastroenterology & Hepatology, Chinese PLA General Hospital, Beijing, China; ^3^ Sidney Kimmel Comprehensive Cancer Center, Johns Hopkins University, Baltimore, Maryland, U.S.A; ^4^ State Key Laboratory of Molecular Oncology, Cancer Institute and Hospital, Chinese Academy of Medical Sciences & Peking Union Medical College, Beijing, China; ^5^ Medical College of NanKai University, Tianjin, China

**Keywords:** DNA methylation, CHFR, esophageal squamous cell cancer, docetaxel, paclitaxel, chemo-sensitivity, 5-aza-2′-deoxycytidine

## Abstract

Esophageal squamous cell carcinoma (ESCC) is one of the most common malignancies worldwide. Both genetic and epigenetic changes are involved in esophageal carcinogenesis. CHFR methylation has been found frequently in different cancers and is regarded as a marker of taxane sensitivity. CHFR methylation was found in 0% (0/16) of normal mucosa, 2.9% (1/34) of grade I dysplasia, 0% (0/8) of grade II dysplasia, 12.5% (1/8) of grade III dysplasia and 45% (49/109) of invasive cancer. When treated with docetaxel or paclitaxel, cell viability was lower in CHFR methylated esophageal cancer cells than in unmethylated cells (p<0.05). No difference was found with either cisplatin or VP16 treatment in either group (p>0.05). In CHFR methylated cells, treatment with docetaxel or paclitaxel resulted in almost all cells being suspended in G0/G1 phase of the cell cycle. After 5-AZ treatment, there was an increased fraction of CHFR-methylated cells in S and G2/M phases (p<0.05). In conclusion, CHFR is frequently methylated in ESCC and the expression of CHFR is regulated by promoter region methylation. CHFR methylation is a late stage event in ESCC. Methylation of CHFR sensitized ESCC cells to taxanes. 5-AZ may re-sensitize chemotherapy resistant in refractory tumors by inducing cell cycle phase re-distribution.

## INTRODUCTION

Esophageal cancer is the eighth most common malignancy and the sixth most frequent cause of cancer-related death worldwide [1, 2]. More than 90 percent of esophageal cancers are either squamous cell or adenocarcinoma in histology [3]. The development of esophageal adenocarcinoma progresses from normal esophageal mucosa through intestinal metaplasia, to low grade and high grade dysplasia, and finally, to invasive cancer [4]. On the other hand, esophageal squamous cell carcinoma (ESCC) develops from normal esophageal epithelium through a hyperproliferative epithelium, to low grade and high grade dysplasia, and finally, to invasive cancer [5, 6]. Although early surgical resection is still the best known approach to improve a patient's survival, most patients are diagnosed with advanced stage where chemotherapy is the most appropriate option. Currently, however, patients' prognosis with chemotherapy remains unsatisfactory because of side effects of chemotherapy and tumor acquired chemo-resistance [7]. Developing an effective chemo-sensitive marker will be a definite advance.

Accumulation of epigenetic changes in tumor suppressor genes is a well known event in human esophageal carcinogenesis [5, 8, 9]. DNA methylation may serve as marker for early cancer diagnosis, chemo-sensitivity as well as prognosis [7, 10-14]. The Checkpoint with forkhead and ring finger domains (CHFR) gene is an early mitotic checkpoint gene that functions as a key player in controlling chromosomal integrity [15]. CHFR knockout mice develop invasive lymphomas as well as solid tumors and are at increased susceptibility to chemical carcinogenesis [16]. Loss of CHFR expression and promoter region hypermethylation were found frequently in different cancers [17-20]. Methylation of CHFR was also regarded as a marker of taxane sensitivity in various cancers [21]. In 35 cases, no significant difference was found in the response of CHFR methylated and unmethylated esophageal cancer patients to the combined cisplatin and 5-fluorouracil therapy plus radiotherapy [22]. The methylation changes of CHFR during carcinogenesis and the sensitivity of esophageal squamous cell carcinoma to taxanes remain unclear.

## RESULTS

### The Expression of CHFR was Silenced by Promoter Region Hypermethylation

**Figure 1:**
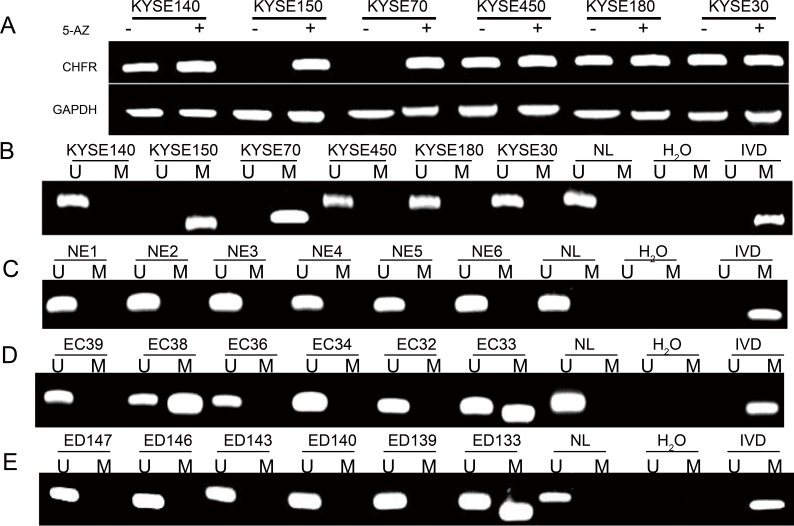
CHFR expression and methylation in esophageal cancer A. The expression of CHFR was examined by RT-PCR. GAPDH: internal control. (−): without 5-AZ treated; (+) 5-AZ treatment . B. CHFR methylation status of human esophageal cancer cell lines. C. CHFR methylation status of human normal esophageal mucosa (NE). D. CHFR methylation status of primary human esophageal cancer(EC). E. CHFR methylation status of human esophageal dysplasia(ED) (U): unmethylated alleles; (M): methylated alleles. (IVD): *in vitro* methylated DNA, served as the methylation control; (NL): normal human peripheral lymphocytes, served as unmethylation control; (H2O): water, served as negative control.

To detect the expression of CHFR in human esophageal cancer, 10 established esophageal cancer cell lines were employed [23]. Constant expression was found in KYSE30, KYSE140, KYSE180, KYSE450, KYSE520, TE7 and SEG1 cells while loss of expression was observed in KYSE70, KYSE150 and KYSE510 cells (Figure 1A). Methylation status was then determined by MSP. Complete methylation was found in KYSE70, KYSE150 and KYSE510 cells while KYSE30, KYSE140, KYSE180, KYSE450, KYSE520, TE7 and SEG1 cells were found to be without methylation (Figure 1B). CHFR expression was found to be inversely correlated to the gene's methylation status. Restoration of CHFR expression was induced by 5-aza-2′-deoxycytidine (5-AZ) treatment in KYSE70, KYSE150 and KYSE510 cells (Figure 1A). These results suggest that the expression of CHFR was regulated by promoter region methylation in esophageal cancer cells.

### CHFR Methylation is a Late Stage Event in the Development of Primary Squamous Esophageal Cell Cancer

Epigenetic alterations have been shown to progress in frequency during esophageal carcinogenesis [5, 6]. To determine if CHFR methylation may serve as an early detection marker of esophageal cancer, we examined the methylation of CHFR in normal esophageal mucosa, different grades of dysplasia, and in invasive cancer. Promoter region hypermethylation was found in 0% (0/16) of normal mucosa, 2.9% (1/34) of grade I dysplasia, 0% (0/8) of grade II dysplasia, 12.5% (1/8) of grade III dysplasia and 44.95% (49/109) of invasive esophageal cancer (Figure 1C,1D,1E, Table1). CHFR was more infrequently methylated in esophageal dysplasia than in invasive esophageal cancer (p<0.01). This suggests that CHFR methylation was a late stage event of esophagus carcinogenesis. No association was found between methylation and age, gender, TNM stage, tumor size, differentiation and lymph node metastasis (all p>0.05).

**Table 1:**
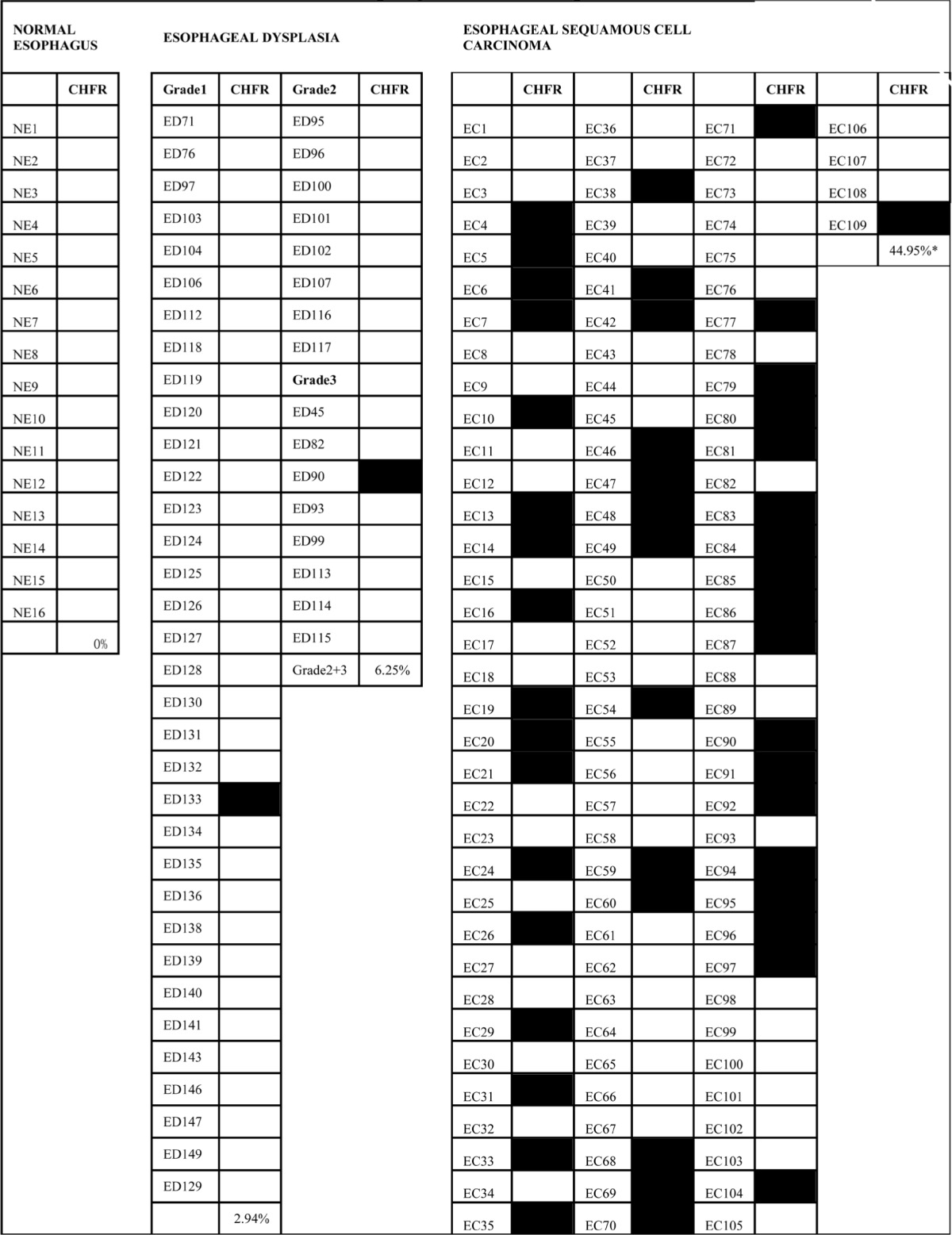
MSP Results of Human Esophageal Tissue Samples

NE=normal esophagus, ED=esophageal dysplasia, EC=esophageal cancer, Black grid= methylated alleles, White grid=unmethylated alleles. CHFR methylation is more frequent in EC than in ED (*P<0.01, χ^2^test ).

### Methylation of CHFR Sensitized Esophageal Cancer Cells to Taxanes

**Figure 2:**
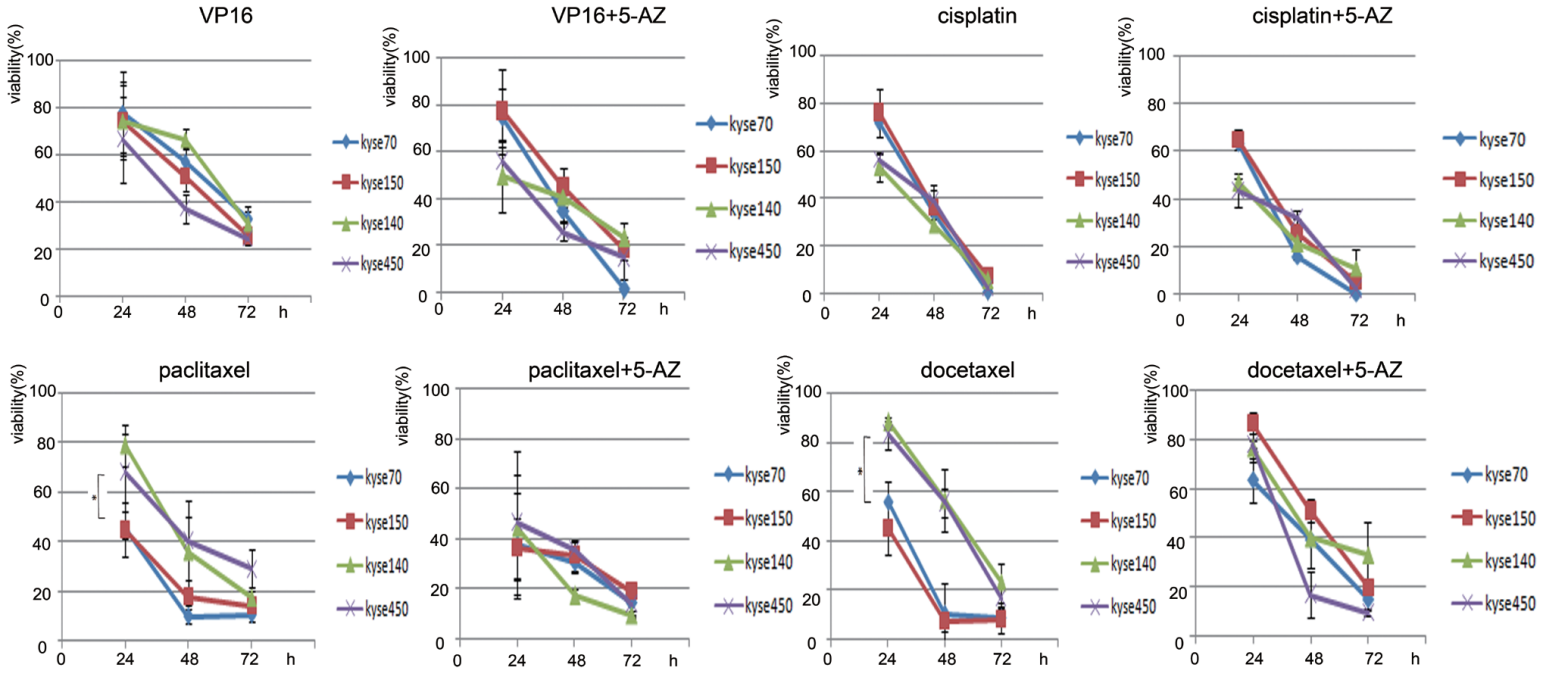
Cell viability in CHFR methylated (M) and unmethylated (U) esophageal cancer cells The viability of CHFR methylated (KYSE70, KYSE150) and unmethylated (KYSE140, KYSE450) cell lines treated by VP16, cisplatin, paclitaxel or docetaxel, with or without 5-AZ treatment at 24hrs, 48hrs or 72hrs as shown in Table 2.

Methylation of CHFR has been shown to sensitize different cancers to taxane treatment [24-26]. In a small study, no association was found between CHFR methylation and the sensitivity of esophageal cancer to cisplatin and 5-fluorouracil [22]. In this study, we compared the methylation status of CHFR and the sensitivity of esophageal cancer cells to paclitaxel, docetaxel, VP16 and cisplatin. No significant difference was found between the viability of unmethylated cells (KYSE140, KYSE450) and methylated cells (KYSE70, KYSE150) in cisplatin or VP16 treatment group (Figure2, table2, all p>0.05). Methylated cells (KYSE70, KYSE150) were significantly more likely to undergo apoptosis than unmethylated cells (KYSE140, KYSE450) after paclitaxel or docetaxel treatment (Figure2, table 2, all p< 0.05). The sensitivity of these CHFR methylated cells was further evaluated with or without 5-AZ treatment. In the cisplatin or VP16 treatment group, cell viability was unchanged with or without 5-AZ treatment (Figure2, table2, all p>0.05). While in the paclitaxel and docetaxel treated groups, no significant difference was found between the methylated (KYSE70, KYSE150) and unmethylated cells (KYSE140, KYSE450) when treated with 5-AZ (Figure2, table2, all p>0.05). These results further suggest that the methylation of CHFR sensitizes esophageal cancer cells to paclitaxel and docetaxel, while the restoration of CHFR expression with 5-AZ induces resistance to these chemotherapeutic agents.

**Table 2:** Cell viability(%) in CHFR methylated (M) and unmethylated (U) esophageal cancer cells

24hrs		VP16 (%)		cisplatin (%)		paclitaxel (%)		Docetaxel (%)	
Cell line	CHFR	5-AZ(−)	5-AZ(+)	5-AZ(−)	5-AZ(+)	5-AZ(−)	5-AZ(+)	5-AZ(−)	5-AZ(+)
KYSE70	M	78.02±17.17	74.57±12.22	72.05±6.29	63.15±1.39	45.07±11.08	37.38±21.10	56.01±7.89	63.27±8.66
KYSE150	M	74.49±15.18	77.27±18.01	76.01±994	64.73±4.37	44.87±3.70	36.33±11.81	44.94±10.53	86.54±4.32
KYSE140	U	74.42±16.43	49.34±14.85	52.97±5.69	46.53±2.35	78.75±8.60	44.34±20.90	88.05±1.74	76.53±6.05
KYSE450	U	66.6±18.12	55.55±9.46	56.12±3.51	43.64±7.14	67.95±15.77	46.4±28.80	83.1± 5.77	77.82±1.39
									
48hrs		VP16 (%)		cisplatin (%)		paclitaxel (%)		Docetaxel (%)	
Cell line	CHFR	5-AZ(−)	5-AZ(+)	5-AZ(−)	5-AZ(+)	5-AZ(−)	5-AZ(+)	5-AZ(−)	5-AZ(+)
KYSE70	M	57.41±5.95	34.7± 4.61	34.19±1.53	15.97±1.06	9.86± 2.68	30.73±4.13	9.94±12.70	39.19±11.63
KYSE150	M	50.88±6.02	44.8± 7.95	36.25±9.66	25.52±6.02	17.63±3.51	33.29±5.76	7.39± 3.92	51.13±4.81
KYSE140	U	66.75±14.85	40.65±4.61	28.94±2.44	21.13±1.59	35.84±14.38	17.16±2.78	56.57±12.57	39.88±12.32
KYSE450	U	37.08±9.46	25.94±3.64	38.99±4.60	31.65±3.65	40.36±15.95	35.53±3.87	55.62±5.71	17± 9.47
									
72hrs		VP16 (%)		cisplatin (%)		paclitaxel (%)		Docetaxel (%)	
Cell line	CHFR	5-AZ(−)	5-AZ(+)	5-AZ(−)	5-AZ(+)	5-AZ(−)	5-AZ(+)	5-AZ(−)	5-AZ(+)
KYSE70	M	32.82±5.24	1.14± 1.63	1.05± 0.88	0.42± 3.25	10.47±2.42	14.73±5.41	8.42± 2.55	14.88±4.45
KYSE150	M	25.45±2.73	18.15±4.29	7.62± 2.60	5.3± 1.07	14.04±2.27	18.96±0.54	8.23± 5.37	20.25±2.93
KYSE140	U	31.08±5.07	22.97±7.03	5.54± 0.93	10.83±7.86	17.2± 2.83	9.77± 1.56	23.12±8.04	32.89±13.36
KYSE450	U	24.63±2.75	14.81±9.24	2.47± 0.58	2.02± 0.91	29.1± 7.60	14.03±4.42	16.85±4.45	9.62± 1.63

VP16, cisplatin, paclitaxel or docetaxel treated cells, with(+) or without(−) 5-AZ treatment in 24hrs, 48hrs or 72hrs. Each experiment was repeated three times.

### The Influence of Docetaxel or Paclitaxel on the Cell Cycle and Apoptosis Before and After 5-AZ treatment in CHFR Methylated Esophageal Cancer Cells

**Figure 3:**
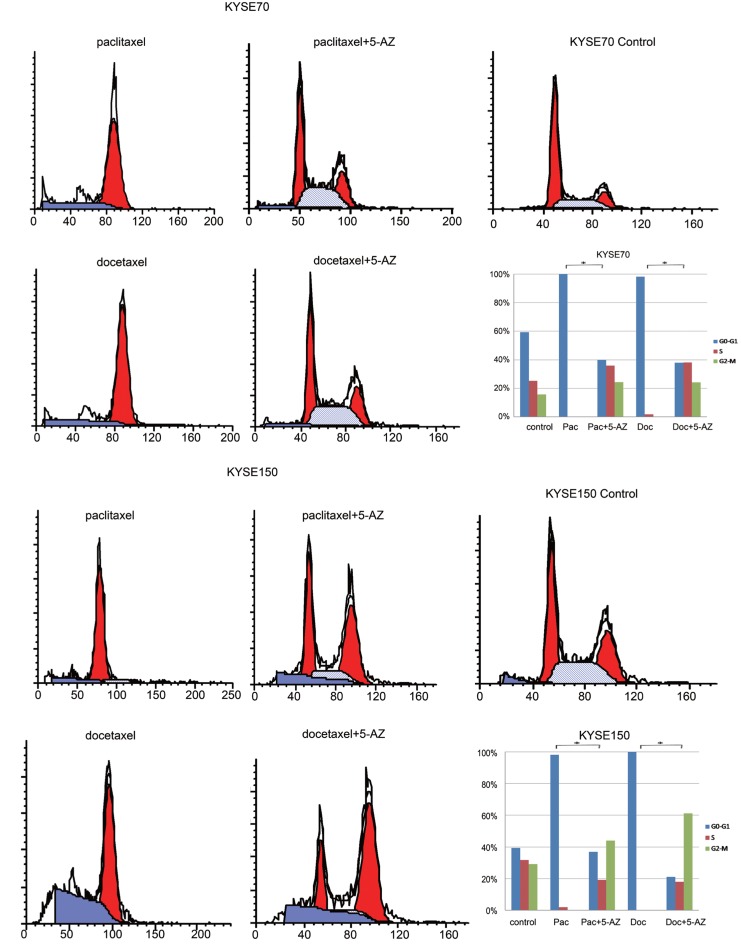
The flow cytometric assay shows the cell phase distribution in methylated esophageal cancer cells (KYSE70 and KYSE150) Pac: paclitaxel; Doc: docetaxel

**Figure 4:**
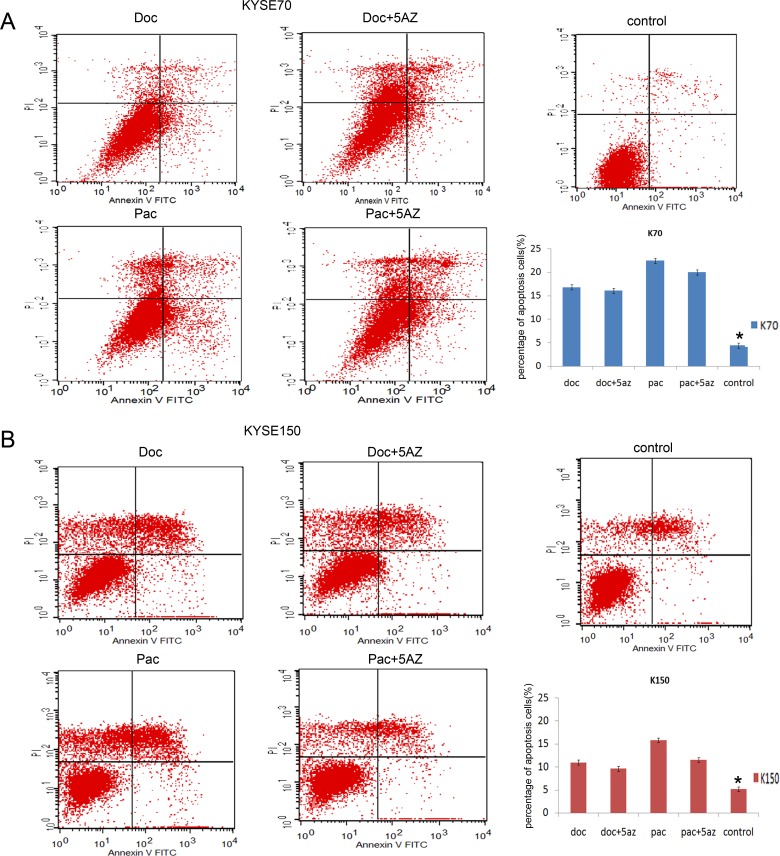
Effect of paclitaxel and docetaxel on cell apoptosis Percentage of KYSE70 and KYSE150 apoptotic cells before and after paclitaxel or docetaxel treatment, with or without 5-AZ treatment

CHFR methylation has been reported to sensitize cancer cells to taxanes. In our study, promoter region methylation sensitized KYSE70 and KYSE150 cells to docetaxel and paclitaxel. To understand the mechanism of promoter region methylation in cheom-sensitivity, we analyzed the cell cycle phase changes in CHFR methylated esophageal cancer cell lines before and after docetaxel/paclitaxel treatment, with or without 5-AZ. In paclitaxel treated KYSE70 cells, cell cycle phase distributions before and after 5-AZ treatment were as follows: 100.00% *vs.* 39.86% in G0/G1 phase, 0.00% *vs.* 35.83% in S phase, 0.00% *vs.* 24.31% in G2/M phase. In docetaxel treated KYSE70 cells, cell cycle phase distributions before and after 5-AZ treatment were as follows: 98.25% *vs.* 37.90% in G0/G1 phase. 1.75% *vs.* 38.04% in S phase, 0.00% *vs.* 24.06% in G2/M phase. In paclitaxel treated KYSE150 cells, cell cycle phase distributions before and after 5-AZ treatment were as follows: 98.11% *vs.* 36.92% in G0/G1 phase, 1.89% *vs.* 19.14% in S phase, 0.00% *vs.* 43.94% in G2/M phase. In docetaxel treated KYSE150 cells, cell cycle phase distributions before and after 5-AZ treatment were as follows: 100.00% *vs.* 21.06% in G0/G1 phase, 0.00% *vs.* 17.85% in S phase, 0.00% *vs.* 61.09% in G2/M phase. In CHFR methylated esophageal cancer cells, under docetaxel or paclitaxel treatment, S and G2/M phase cells were increased with the treatment of 5-AZ (Figure3, all p<0.05). These results may partially explain the mechanism by which 5-AZ can overcome chemotherapy resistance in refractory solid tumors [27]. Apoptosis was analyzed before and after docetaxel/paclitaxel treatment in KYSE70 and KYSE150 cells. The ratio of apoptosis was 16.78 ± 5.42% *vs.* baseline of 4.39 ± 0.56% and 22.45 ± 3.49% *vs.* 4.39 ± 0.56% for docetaxel or paclitaxel ( both P<0.05) in KYSE70 cells. In KYSE150 cells, the apoptotic rate was 10.97 ± 0.61% *vs.* baseline of 5.16 ± 0.51% and 15.78 ± 1.09 *vs*.5.16 ± 0.51% for docetaxel or paclitaxel (all P<0.05) (Fig.4). No significant changes were found between the docetaxel/paclitaxel treatment groups and the docetaxel/paclitaxel combined with 5-AZ treatment groups.

## DISCUSSION

Accumulation of genetic and epigenetic changes has been found in the development of various cancers, including esophageal cancer [28-30]. The methylation status during the carcinogenesis of esophageal squamous cell, however, remains unclear. In this study, we found that CHFR is infrequently methylated early in the esophageal carcinogenesis, but becomes more frequently methylated during the advanced cancer stage. It suggests that CHFR methylation is a late stage marker of esophageal squamous cell carcinoma. Methylation of CHFR has been regarded as a chemo-sensitive marker in various cancers [24-26]. We found CHFR methylation sensitizes esophageal cancer cells to docetaxel and paclitaxel, and 5-AZ induces chemoresistance of CHFR methylated cells to docetaxel and paclitaxel. These results suggest that CHFR methylation is a taxane sensitive marker in human esophageal squamous cancer. There are a few reports about the ability of epigenetic therapy to undo chemoresistance and re-sensitize refractory tumors to specific chemotherapy agents [27, 31-33]. But the definitive mechanism remains to be elucidated. To answer this question, we analyzed cell cycle distributions in docetaxel and paclitaxel treated esophageal cancer cells with or without 5-AZ treatment. After docetaxel or paclitaxel treatment, almost all viable cells were in G0/G1 phase. When docetaxel or paclitaxel is combined with 5-AZ treatment, S and G2/M phase cells were increased. This result may be interpreted as a partial explanation for the mechanism by which epigenetic therapy re-sensitizes chemotherapy resistant, refractory tumors. Microtubule inhibitors, such as docetaxel and paclitaxel, disrupt normal microtubule dynamics during cell division by binding to the beta-tubulin subunits. This can lead to a failure of microtubule separation and apoptosis. As CHFR is able to block entry into prophase until chromosomal alignment is restored, CHFR inhibits the effect of taxanes [21, 34]. Accordingly, cells expressing CHFR are more viable upon treatment with microtubule inhibitors compared to cells not expressing CHFR [21, 34]. In our study, silencing CHFR by promoter region hypermethylation sensitized esophageal cancer cells to docetaxel and paclitaxel. In addition, CHFR expression was induced by 5-AZ in CHFR methylated esophageal cancer cells. We also observed that S and G2/M phase cells were increased in docetaxel and paclitaxel treated esophageal cancer cells when combined with 5-AZ treatment. The ability of 5-AZ to re-sensitize refractory tumors to chemotherapy may be due to the ability of 5-AZ in some cancers to change the cell cycle phase.

In conclusion, CHFR is frequently methylated in human esophageal squamous cell carcinoma and the expression of CHFR is regulated by promoter region methylation. Methylation of CHFR is a late stage event in esophageal cancer. Methylation of CHFR sensitized esophageal cancer cells to docetaxel and paclitaxel. Finally, 5-AZ may re-sensitize chemotherapy resistant refractory tumors by inducing cell cycle phase re-distribution.

## MATERIAL & METHODS

### Human tissue samples and cell lines

Ten human esophageal cancer cell lines (KYSE30, KYSE140, KYSE180, KYSE450, KYSE520, TE7, SEG1, KYSE70, KYSE150 and KYSE510) were used in this study [23]. All the esophageal cancer cell lines were established from primary esophageal cancer and maintained in 90% RPMI 1640 (Invitrogen, CA, USA), supplemented with 10% fetal bovine serum and antibiotics. Sixteen cases of normal esophageal mucosa and 109 cases of invasive esophageal squamous cancer specimens were collected as fresh frozen tissue from the Chinese General PLA Hospital and kept at −80°C. All tissue samples were classified by TNM staging, stage I (n=2), stage II (n=65), stage III (n=41) and stage IV (n=1). Themedian age of the patients was 59.35 ± 8.91. Fifty cases of paraffin-embedded esophageal squamous dysplasia samples, including grade 1 (n=34), grade 2 (n=8), and grade 3 (n=8) specimens, were collected from the Second Teaching Hospital, Zhengzhou University. All tissues sample were collected according to the approved guidelines of the Institutional Review Boards of both the Chinese PLA General Hospital and the Second Teaching Hospital, Zhengzhou University.

### 5-aza-2′-deoxycytidine (5-AZ) treatment

Esophageal cancer cells were split to a low density (30% confluence) 12 h before treatment. Cells were treated with 5-AZ (Sigma, St. Louis, MO, USA) at a concentration of 2 μM in the growth medium, which was exchanged every 24 h for a total of 96 h treatment. At the end of the treatment course, RNA was isolated as described below.

### RNA isolation and semi-quantitative RT-PCR

Total RNA was isolated by Trizol reagent (Invitrogen, Carlsbad, USA). RNA quality and quantity were evaluated by 1% Agarose gel electrophoresis and spectrophotometric analysis. Semi-quantitative reverse transcription-PCR (RT-PCR) was performed as described previously[35]. RT-PCR primers were as follows: 5′-GGCGAGAGCGTTCCTCCAGTTG-3′ (F); 5′-GCATGTCAGCGTCTCCTCCATCTTG-3′(R).

### DNA Extraction, Methylation-Specific PCR (MSP)

Genomic DNA of esophageal cancer cells and tissue samples were extracted by the proteinase K method. MSP was performed as described previously [5, 36, 37]. Primers for MSP were designed around the transcription start site. The sequences of the MSP primers were as follows: 5′-GATTGTAGTTATTTTTGTGATTTGTAGGTGAT-3′ (UF), 5′-AACTAAAACAAAACCAAAAATAACCCACA-3′ (UR), 5′-GTTATTTTCGTGATTCGTAGGCGAC-3′ (MF) and 5′-CGAAACCGAAAATAACCCGCG-3′ (MR). The amplification was performed as follows: an initial step at 95°C for 5min; then 35 cycles of 95°C for 30s, 60°C for 30s and 72°C for 30s; the final extension step at 72°C for 5min.

Meanwhile, for paraffin-embedded esophageal dysplasia, we first performed a NESTED-PCR, and then carried out MSP using the NESTED-PCR product. External primer sequences were: CHFR Flank-F: 5′-TTTTYGTTTTTTTTGTTTTAATATAATATGG-3′ CHFR Flank-R: 5′-CRCRCACCAAAAACRACAACRAAAAC-3′. The PCR products were then diluted with H O to 1:1,000 and 2ul was used as template for each 25 ul reaction MSP analysis. PCR cycle conditions were as follows: 95°C for 5min; then 30 cycles of 95°C for 30s, 60°C for 30s and 72°C for 30s; the final extension step at 72°C for 5min.

### Cell viability assay

KYSE70, KYSE 150, KYSE 140 and KYSE 450 were suspended at 5 × 103 cells per well and subcultured in a 96-well plate. These cell lines were prepared in two groups. All four cell lines were included in each group and each group had three time points (24 h, 48 h and 72 h). After passage for 16 h, group 1 was treated with 10 μg/mL paclitaxel (Pac), 18.4 μg/ml docetaxel (Doc), 25 μg/ml VP16, or 10μg/ml cisplatin for 24 h, 48 h and 72 h respectively. Group 2 was treated with 2μmol/L 5-AZ+10μg/ml paclitaxel, 5-AZ+18.4 μg/ml docetaxel (Doc), 5-AZ+ 25 μg/ml VP16, or 5-AZ+10 μg/ml cisplatin for 24 h, 48 h and 72 h respectively. Cell viability was measured at 24, 48 and 72 h using the Dojindo Cell Counting Kit-8(CCK8 kit, Dojindo Laboratories, Gaithersburg, MD) according to the company's instruction. Absorbance was measured on a microplate reader (Thermo Multiskan MK3 USA) at a wave length of 450 nm.

### Flow Cytometry Analysis of Cell Cycle and Apoptosis

KYSE70, KYSE150 cells were suspended at 5 × 103 cells in a 24-well plate to 80% confluence. RPMI1640 +10% FBS medium were used as controls. The treatment groups were treated for 24 h with RPMI1640 +10% FBS medium including 0.1μg/ml Pac, 0.1μg/ml Pac +12μmol/L 5-AZ, 0.2μg/mL Doc, 0.2μg/mL Doc+12μmol/L 5-AZ, respectively.

Cell cycle was analyzed as described previously [36]. Cells were harvested, fixed in 75% ethanol, incubated with 100μg /ml RNase, and stained in 50 μg/ml PI solution. The PI fluorescence was then measured using an EpicsXL MCL flow cytometer (Beckman Coulter Inc., Fullerton, CA, USA). The data were analyzed using Cell Quest software (Becton Dickinson Biosciences).

For apoptosis analysis, Annexin V-FITC/PI Apoptosis Detection Kit (KeyGen Biotechnology, China) was utilized according to the manufacturer's instructions.

### Statistical Analysis

The results are expressed as mean ± standard deviation (SD). Statistical analysis was performed with SPSS17.0 software for multiple comparisons. χ2 test and student's test were used. P < 0.05 was considered to be statistically significant.
